# miR-186-5p调控PRKAA2促进肺腺癌细胞铁死亡的机制研究

**DOI:** 10.3779/j.issn.1009-3419.2023.102.39

**Published:** 2023-11-20

**Authors:** Lu LIU, Xin GUAN, Yanqiao ZHAO, Xiaona WANG, Chonggao YIN, Qinghua LIU, Hongli LI

**Affiliations:** ^1^261053 潍坊，潍坊医学院病理学教研室; ^1^Department of Pathhology, Weifang Medical University, Weifang 261053, China; ^2^261053 潍坊，临床医学院; ^2^Clinical Medical College, Weifang Medical University, Weifang 261053, China; ^3^261053 潍坊，护理学院; ^3^Colloge of Nursing, Weifang Medical University, Weifang 261053, China; ^4^261053 潍坊，基础医学院; ^4^College of Basic Medical Sciences, Weifang Medical University, Weifang 261053, China; ^5^261053 潍坊，医学研究实验中心; ^5^Medical Research Center, Weifang Medical University, Weifang 261053, China

**Keywords:** 肺肿瘤, miR-186-5p, PRKAA2, 增殖, 侵袭, 迁移, 铁死亡, Lung neoplasms, miR-186-5p, PRKAA2, Proliferation, Invasion, Migration, Ferroptosis

## Abstract

**背景与目的:**

肺腺癌是最常见的非小细胞肺癌组织学类型，miRNAs作为基因表达的调控分子调控细胞增殖、凋亡和分化，从而影响细胞内稳态。本研究通过实验验证miR-186-5p通过调控其下游靶蛋白PRKAA2影响铁死亡途径从而抑制肺腺癌细胞增殖、迁移和侵袭能力。

**方法:**

前期研究获得肺癌中表达显著下调miRNA，即miR-186-5p；生物信息学方法预测其下游铁死亡相关靶蛋白并查询其在肺腺癌中的表达水平及对患者生存预后的影响；双荧光素酶验证两者结合位点；实时荧光定量聚合酶链式反应（quantitative real-time polymerase chain reaction, qRT-PCR）和Western blot检测过表达miR-186-5p后PRKAA2的基因和蛋白表达情况；EdU、Transwell、划痕实验验证miR-186-5p在A549、H1299细胞中的增殖、侵袭迁移能力的影响以及miR-186-5p对Fer-1抑制铁死亡敏感性的作用机制；活性氧（reactive oxygen species, ROS）实验检测miR-186-5p对肺腺癌细胞ROS含量的影响；丙二醛（malondialdehyde, MDA）、谷胱甘肽（glutathione, GSH）实验检测miR-186-5p、PRKAA2对肺腺癌细胞铁死亡指标变化的影响；脂质ROS（lipid ROS, L-ROS）实验检测miR-186-5p、PRKAA2对肺腺癌细胞L-ROS含量的影响。

**结果:**

PRKAA2在肺腺癌中表达上调；过表达miR-186-5p使PRKAA2的基因和蛋白表达量下降；过表达miR-186-5p通过调控PRKAA2促进铁死亡抑制肺腺癌细胞增殖、侵袭迁移能力；miR-186-5p能够增加A549细胞ROS含量；过表达miR-186-5p和敲低PRKAA2上调肺腺癌细胞MDA含量，下调还原型GSH含量；miR-186-5p通过靶向PRKAA2增加肺腺癌细胞L-ROS含量，促进肺腺癌细胞铁死亡敏感性。

**结论:**

miR-186-5p通过靶向调控PRKAA2促进肺腺癌A549、H1299细胞铁死亡，从而抑制肺腺癌增殖、侵袭和迁移能力。

## Abstract (en)

**Background and objective:**

Lung adenocarcinoma (LUAD) is the most common type of non-small cell lung cancer, and any change of miRNAs expression will affect the degree of target regulation, thus affecting intracellular homeostasis. This study verified that miR-186-5p could inhibit the proliferation, migration and invasion of LUAD cells by regulating PRKAA2.

**Methods:**

Previous investigations found that the expression of miR-186-5p was markedly suppressed in LUAD. Bioinformatics method is used to predict the target protein related to ferroptosis downstream and inquire about its expression level in LUAD and its influence on the survival of patients. Double luciferase verified the binding site of PRKAA2 and miR-186-5p. Quantitative real-time polymerase chain reaction (qRT-PCR) and Western blot were used to detect the expression of PRKAA2. The effects of miR-186-5p of LUAD cells as well as the mechanism by which miR-186-5p inhibits Fer-1's sensitivity to ferroptosis were confirmed by EdU, Transwell, and scratch assays. The effect of miR-186-5p on the amount of reactive oxygen species (ROS) in LUAD cells was discovered using ROS experiment. Malondialdehyde (MDA) and glutathione (GSH) experiments were used to detect the effects of miR-186-5p and PRKAA2 on ferroptosis index of LUAD cells. The concentration of lipid ROS (L-ROS) in LUAD cells were measured using the L-ROS tests to determine the effects of miR-186-5p and PRKAA2.

**Results:**

The expression of PRKAA2 is up-regulated, and a high level of PRKAA2 expression was associated with a poor prognosis for patients with LUAD. Overexpression of miR-186-5p decreased the gene and protein expression of PRKAA2. By promoting ferroptosis, miR-186-5p overexpression prevented lung cancer cells from proliferating, invading, and migrating. ROS could be produced in higher amounts in LUAD cells due to miR-186-5p. Overexpression of miR-186-5p and knockdown PRKAA2 up-regulated MDA content and reduced GSH content in LUAD cells, respectively. miR-186-5p could increase the content of L-ROS and promote the ferroptosis sensitivity of LUAD cells by targeting PRKAA2.

**Conclusion:**

miR-186-5p promotes ferroptosis of LUAD cells through targeted regulation of PRKAA2, thus inhibiting the proliferation, invasion and migration of LUAD.

肺癌是全球癌症相关死亡的主要原因^[1]^，肺腺癌（lung adenocarcinoma, LUAD）是最常见的肺癌组织学类型^[2]^，肺癌患者的平均5年生存率仍然很低，致癌基因的有效监视被视为癌症治疗的靶点，分子病理学诊断和靶向治疗应用能显著提高患者总生存期^[3]^。目前分子靶向治疗在临床应用中显示出不错的治疗效果^[4]^，但是由于耐药性的发展，患者的治疗仍具有挑战性。因此，进一步了解肺癌发生和发展的分子机制，寻找更有效的分子靶向治疗和预后预测指标对提高LUAD患者的生存率和生活质量至关重要^[5]^。

铁死亡（ferroptosis）是一种新的细胞死亡形式，其特征是大量铁积累和脂质过氧化，在肿瘤微环境中起着关键作用^[6]^。铁稳态影响细胞特征的发展，细胞铁含量可以影响肿瘤状况，肿瘤细胞的生长和增殖需要大量的铁，但过量的铁水平会导致肿瘤细胞死亡^[7]^。先前研究证实，LUAD中重要的铁死亡调节因子RRM2通过抑制铁毒死亡促进肿瘤免疫浸润^[8]^；KLF11通过抑制GPX4调控LUAD铁下垂和化疗敏感性^[9]^，因此解决抑制铁死亡的分子机制、诱导铁死亡发生对于治疗肺癌具有深远临床意义。

微小RNA（microRNAs, miRNAs）是一种高度保守的非编码RNA（non-coding RNAs, ncRNAs）^[10]^，主要通过3' UTR中的识别位点进行翻译抑制或降解信使RNA（messenger RNA, mRNA）来调节基因的稳定性表达^[11]^。有课题组研究^[12]^表明，lncRNA-AC009948.5通过结合miR-186-5p促进LUAD的侵袭转移，miR-186-5p降低A549细胞的迁移和侵袭能力，miR-186-5p可能成为LUAD诊断和预后的生物标志物，但关于miR-186-5p是否通过铁死亡方式影响LUAD增殖、侵袭与迁移还尚未见研究与报道。本文通过细胞功能实验验证miR-186-5p是否通过调控其下游靶蛋白PRKAA2影响A549铁死亡途径从而抑制LUAD细胞增殖、迁移和侵袭能力。

## 1 资料与方法

### 1.1 主要试剂与仪器

细胞株：LUAD A549、H1299细胞株以及293T细胞株购自ATCC（American Type Culture Collection）。miR-186-5p过表达及对照质粒、PRKAA2敲除及对照质粒均由上海吉凯基因股份有限公司构建合成。铁死亡抑制剂Fer-1购自山东增峰生物科技有限公司。EdU和JC-1试剂盒购自碧云天生物技术有限公司。活性氧（reactive oxygen species, ROS）、丙二醛（malondialdehyde, MDA）、谷胱甘肽（glutathione, GSH）试剂盒购自Boxbio生工科技有限公司。丝裂霉素C购自Sigma Aldrich公司。

### 1.2 细胞转染与分组

将细胞分组：（1）con组：A549、H1299细胞转染对照质粒；（2）con+Fer-1组：转染对照质粒后加入10 μmol/L铁死亡抑制剂Fer-1；（3）over-miR-186-5p+Fer-1组：转染过表达miR-186-5p质粒后加入10 μmol/L铁死亡抑制剂Fer-1；（4）over-miR-186-5p+sh-PRKAA2+Fer-1组：共转染过表达miR-186-5p和敲低PRKAA2质粒后加入10 μmol/L铁死亡抑制剂Fer-1。

### 1.3 在线网站预测和筛选靶基因

通过miRWalk数据库、DIANA数据库（https://diana.e-ce.uth.gr/tools）、miRDB数据库（http://mirdb.org/）以及FerrDb数据库（http://www.zhounan.org/ferrdb/current/）预测可能与miR-186-5p结合的靶基因。通过韦恩图将以上网站所预测到的靶蛋白取交集。通过GEPIA和UALCAN数据库最终筛选出表达以及生存预后符合条件的靶蛋白。

### 1.4 双荧光素酶报告基因检测

构建pGL3-PRKAA2-3’-UTR-WT（UGAAAA UCAGUUAUAUUCUUUA）和pGL3-PRKAA2-3’-UTR-MUT（UGAAAA UCAGUUAUCGCGAUCA），将miR-186-5p过表达（CTCGAGTG CTTGTAACTTTCC AAAGAATTCTCCTTTTGGGCTT TCTGGTTTTATT TTAAGCCCAAAGGTGAATTTTTTGGG AAG TTTGAGCTTTCGAA）及对照质粒和PRKAA2的野生型质粒、突变型质粒用Lipofectamine 2000共转染入293T细胞，48 h后测定荧光素酶活性。

### 1.5 实时荧光定量聚合酶链式反应（quantitative real-time polymerase chain reaction, qRT-PCR）检测PRKAA2基因表达水平

细胞转染24 h后，提取细胞总RNA，逆转录为互补DNA（complementary DNA, cDNA），qRT-PCR检测PRKAA2在细胞中的表达量。

### 1.6 Western blot实验检测PRKAA2蛋白表达水平

细胞转染后提取总蛋白，煮蛋白，电泳，转膜，封闭，一抗4 ^o^C过夜，洗膜，二抗孵育，洗膜，曝光。实验重复3次。

### 1.7 EdU细胞增殖实验

各分组细胞转染24 h后，加入EdU工作液，37 ^o^C孵育2 h，甲醇固定30 min，Triton透膜40 min，Click反应液孵育40 min，DAPI染核。

### 1.8 Transwell侵袭实验

取培养24 h的各组转染细胞，无血清培养基重悬。取稀释后的Matrigel胶涂抹于上室。上室滴加200 μL（4×10^4^个）细胞悬液，下室滴加含10% FBS的正常培养液。24 h后甲醇固定，PBS洗涤，姬姆萨染色，双蒸水冲洗晾干，拍照并计数。实验独立重复3次。

### 1.9 划痕愈合实验

取移液器200 μL吸头做平行划痕，PBS洗涤，更换为含1%胎牛血清的培养基。独立实验重复3次。

### 1.10 MDA和还原型GSH含量检测

各组转染细胞数量1×10^4^个，离心收集细胞，冰浴超声破碎，4 ^o^C 8000 g离心10 min。取上清加入MDA检测试剂后沸水浴处理60 min，测定450、532和600 nm三处的吸光值进行MDA含量计算；另取上清加入GSH检测试剂后室温充分显色，酶标仪测定412 nm处吸光值进行GSH含量计算。

### 1.11 ROS和脂质活性氧（lipid ROS, L-ROS）含量检测

各组转染过的细胞以2×10^5^数量铺于共聚焦小皿中，加入无血清培养液稀释DCFH-DA荧光探针的工作液（1:1000，终浓度10 μmol/L），避光孵育20 min；无血清培养基洗涤后换为正常培养液，观察并拍照。L-ROS：甲醇固定20 min，PBS洗涤，加入1 mL用无血清培养液稀释C11-BODIPY探针的工作液，孵育30 min，PBS洗涤，换为正常培养液。观察并拍照，实验重复3次。

### 1.12 统计学方法

采用SPSS 23.0进行统计学分析处理，数据均用均数±标准差（Mean±SD）表示，两组样本比较采用t检验，多组样本比较采用单因素方差分析，P<0.05被认为差异具有统计学意义。

## 2 结果

### 2.1 PRKAA2与miR-186-5p靶向结合且在LUAD中高表达

通过生信网站miRDB、DIANA、miRWalk、FerrDb预测可与miR-186-5p结合的铁死亡相关靶基因，4组数据取交集得到2个靶基因：PIK3CA、PRKAA2（图1A）。通过GEPIA和Ualcan数据库对预测到的靶蛋白进行分析发现，PRKAA2在LUAD组织中高表达（图1B，P<0.05），PRKAA2低表达的LUAD患者预后生存率高于高表达患者（图1C，P<0.05）。因此，我们选取PRKAA2为研究对象做后续研究。双荧光素酶实验验证miR-186-5p与PRKAA2两者间靶向结合关系，结果显示，PRKAA2 mRNA的3’-UTR区是miR-186-5p的直接结合位点（图1D，P<0.05）。综上所述，PRKAA2在LUAD中高表达，miR-186-5p与PRKAA2存在结合位点且miR-186-5p负向调控PRKAA2蛋白表达。

**图1:**
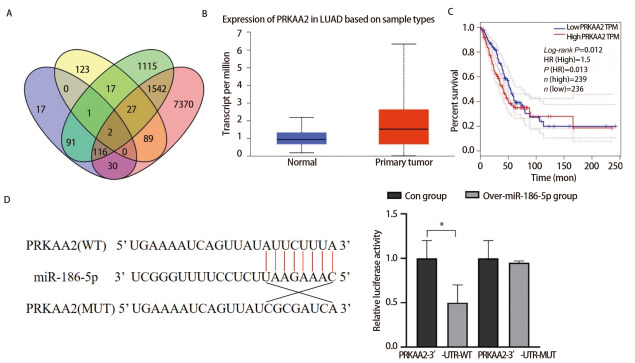
miR-186-5p与PRKAA2靶向结合。A：韦恩图显示4个生信网站取交集获得2个共同铁死亡蛋白；B：Ualcan查询PRKAA2在正常组织和肺腺癌组织中的表达情况；C：GEPIA查询PRKAA2的生存曲线分析；D：荧光素酶实验检测不同组细胞的荧光素酶活性。*P<0.05。

### 2.2 miR-186-5p负向调控PRKAA2发挥作用

通过qRT-PCR检测PRKAA2的基因表达水平。结果显示与对照组相比，过表达miR-186-5p后PRKAA2表达量显著下降（图2A）；通过Western印迹检测过表达miR-186-5p后PRKAA2蛋白的表达情况。结果显示，over-miR-186-5p组PRKAA2的表达明显低于con组（图2B，P<0.05）。综上所述，miR-186-5p负向调控PRKAA2从而发挥作用。

**图2:**
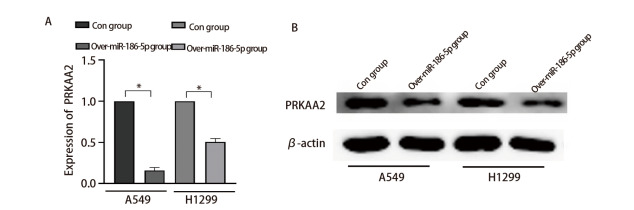
miR-186-5p负向调控PRKAA2。A：qRT-PCR检测过表达miR-186-5p后PRKAA2的基因表达水平；B：Western blot检测过表达miR-186-5p后PRKAA2的蛋白表达水平。*P<0.05。

### 2.3 miR-186-5p通过铁死亡方式抑制LUAD细胞增殖能力

EdU增殖实验结果显示，在相同浓度（10 μmol/L）抑制剂Fer-1处理下，con+Fer-1组阳性增殖细胞数明显多于con组；相较于con+Fer-1组，over-miR-186-5p+Fer-1组A549和H1299阳性细胞数相对减少；over-miR-186-5p+sh-PRKAA2+Fer-1组阳性增殖细胞数比over-miR-186-5p+Fer-1组更少（图3A、3B），差异具有统计学意义（P<0.05）。

**图3:**
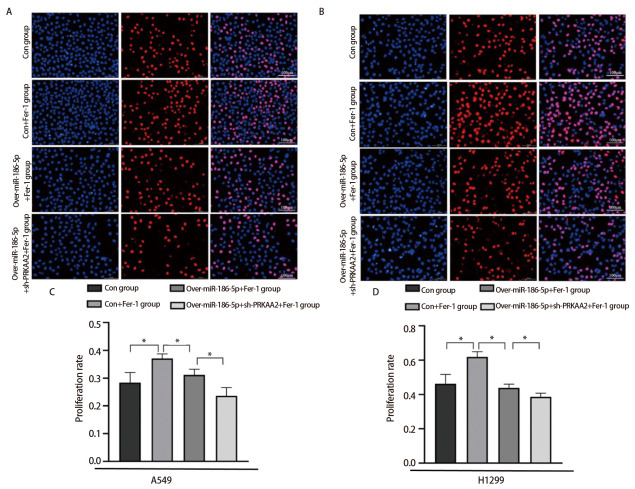
miR-186-5p调控PRKAA2通过铁死亡方式抑制肺腺癌细胞增殖能力。A：EdU实验检测在加入Fer-1条件下，转染miR-186-5p和PRKAA2对A549细胞增殖能力的结果图及定量分析；B：相同条件下对H1299细胞侵袭能力的结果图及定量分析。*P<0.05。

### 2.4 miR-186-5p通过铁死亡方式抑制LUAD细胞侵袭迁移能力

Transwell侵袭实验结果显示（图4），在相同浓度（10 μmol/L）抑制剂Fer-1处理下，con+Fer-1组穿过基质胶的细胞数明显多于con组，相较于con+Fer-1组，over-miR-186-5p+Fer-1组穿过基底膜的A549和H1299细胞数相对减少；over-miR-186-5p+Fer-1组穿过基底膜的细胞数多于over-miR-186-5p+sh-PRKAA2+Fer-1组。划痕愈合实验结果显示，每组在加入丝裂霉素C排除细胞增殖对划痕迁移能力的影响后，在相同浓度（10 μmol/L）抑制剂Fer-1处理下，相较于con组，con+Fer-1组划痕宽度更窄；over-miR-186-5p+Fer-1组细胞迁移率小于con+Fer-1组；相较于over-miR-186-5p+Fer-1组，over-miR-186-5p+sh-PRKAA2+Fer-1组划痕宽度更加显著（图5A、5B），差异具有统计学意义（P<0.05）。

**图4:**
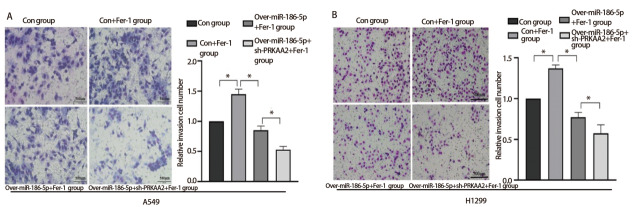
miR-186-5p调控PRKAA2通过铁死亡方式抑制肺腺癌细胞侵袭能力。A：Transwell实验检测在加入Fer-1条件下，转染miR-186-5p和PRKAA2对A549细胞侵袭能力的结果图及定量分析；B：相同条件下对H1299细胞侵袭能力的结果图及定量分析。*P<0.05。

**图5:**
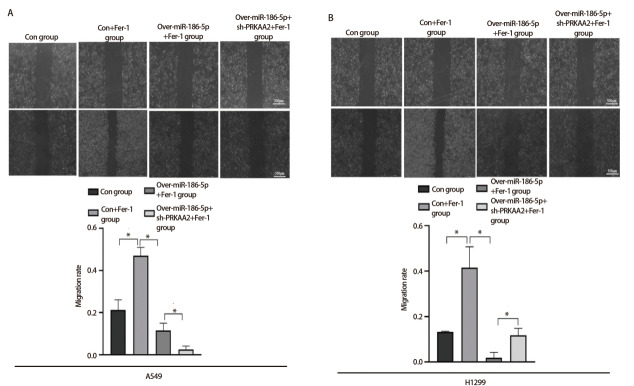
miR-186-5p调控PRKAA2通过铁死亡方式抑制肺腺癌细胞迁移能力。A：划痕实验检测在加入Fer-1条件下，转染miR-186-5p和PRKAA2对A549细胞迁移能力的结果图及定量分析；B：相同条件下对H1299细胞侵袭能力的结果图及定量分析。*P<0.05。

### 2.5 miR-186-5p逆转Fer-1减少LUAD细胞ROS含量的影响

ROS实验结果显示，在相同浓度（10 μmol/L）抑制剂Fer-1处理下，con+Fer-1组细胞荧光强度明显低于con组；相较于con+Fer-1组，over-miR-186-5p+Fer-1组A549和H1299细胞荧光强度相对增强；over-miR-186-5p+sh-PRKAA2+Fer-1组荧光强度强于over-miR-186-5p+Fer-1组，过表达miR-186-5p和敲低PRKAA2引起大量ROS的生成（图6），差异具有统计学意义（P<0.05）。

**图6:**
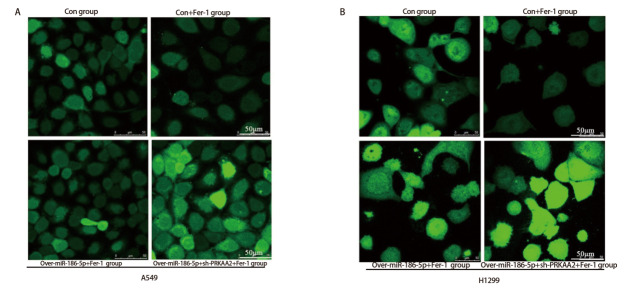
ROS实验检测各组细胞内ROS含量变化。敲低PRKAA2协同miR-186-5p共同升高细胞内ROS含量。

### 2.6 miR-186-5p调控PRKAA2通过调节MDA与GSH含量促进铁死亡敏感性

MDA实验显示，与con组相比，con+Fer-1组细胞MDA含量降低；相较于con+Fer-1组，over-miR-186-5p+Fer-1组A549和H1299细胞MDA含量升高；over-miR-186-5p+sh-PRKAA2+Fer-1组细胞MDA含量显著高于over-miR-186-5p+Fer-1组。GSH实验结果显示，与con组相比，con+Fer-1组还原型GSH含量升高；相较于con+Fer-1组，over-miR-186-5p+Fer-1组A549和H1299细胞还原型GSH含量降低；over-miR-186-5p+sh-PRKAA2+Fer-1组细胞还原型GSH含量显著低于over-miR-186-5p+Fer-1组（图7）。因此，过表达miR-186-5p逆转了Fer-1对LUAD细胞铁死亡指标的影响，过表达miR-186-5p和敲除PRKAA2可明显上调A549和H1299细胞中MDA水平，下调GSH含量，差异具有统计学意义（P<0.05）。

**图7:**
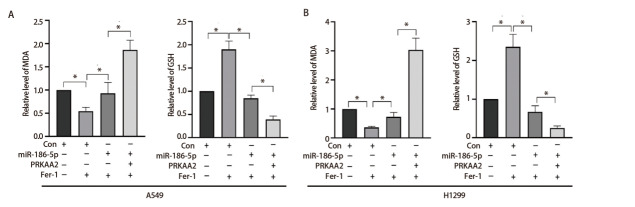
miR-186-5p和PRKAA2对A549（A）、H1299（B）细胞MDA和GSH水平的影响。*P<0.05。

### 2.7 miR-186-5p调控PRKAA2通过增加L-ROS促进铁死亡敏感性

采用C11 BODIPY 581/591检测A549脂质过氧化情况，未氧化的C11 BODIPY 581/591呈现红色荧光信号，氧化的呈绿色荧光信号。L-ROS实验结果显示，相较于con组，con+Fer-1组A549和H1299细胞L-ROS红色荧光信号强度升高，over-miR-186-5p+Fer-1组A549细胞红色荧光强度减弱；相较于over-miR-186-5p+Fer-1组，over-miR-186-5p+sh-PRKAA2+Fer-1组细胞绿色荧光信号显著增强，红色荧光明显减弱（图8），miR-186-5p通过靶向调控PRKAA2增强L-ROS水平，打破细胞内氧化还原平衡，诱导A549和H1299细胞L-ROS生成，差异具有统计学意义（P<0.05）。

**图8:**
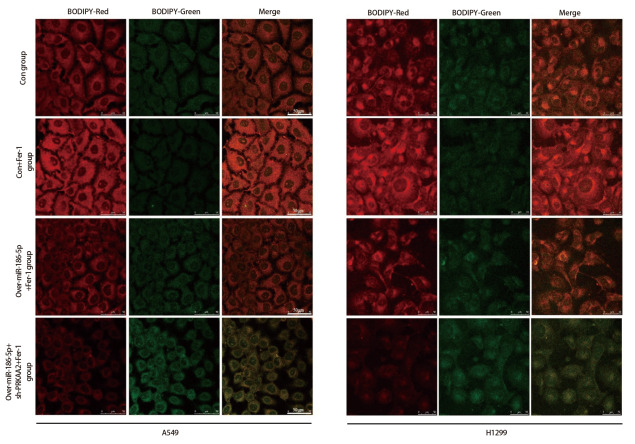
miR-186-5p靶向PRKAA2破坏A549、H1299细胞中的铁稳态。L-ROS实验检测在加入Fer-1条件下，过表达miR-186-5p和敲低PRKAA2后细胞内L-ROS含量变化。

## 3 讨论

LUAD常见于肺外周组织，约占非小细胞肺癌（non-small cell lung cancer, NSCLC）的70%和肺肿瘤的40%。针对性地靶向打击癌细胞是癌症治疗的主要挑战，癌症的发生和维持均有癌基因的调控，因此抑制肿瘤生长的策略之一是靶向致癌途径^[13]^。随着精准医疗的快速发展，人们提出了新的治疗策略，以改善LUAD患者的预后^[14]^。确定肺癌发生发展机制是开发有效的生物标志物，以更好地诊断和设计治疗干预措施的必要条件^[15]^。

miR-186-5p参与多种形式的细胞死亡过程，如凋亡、焦亡和自噬。本研究发现miR-186-5p通过结合PRKAA2抑制Fer-1促进A549、H1299细胞铁死亡，这些研究表明miR-186-5p是介导细胞凋亡、焦亡、自噬和铁死亡交叉调控的重要miRNA，进一步探讨miR-186-5p在细胞凋亡、自噬和铁死亡之间相互作用中的关键作用具有深远的临床意义。铁对于快速增殖的癌细胞至关重要，已经有人尝试通过铁代谢来抑制肿瘤生长^[16]^。上皮-间充质转化（epithelial-mesenchymal transition, EMT）赋予极化上皮细胞更强的侵袭性，被认为是肿瘤转移的关键步骤^[17]^。铁下垂和EMT是肿瘤发生的主要生物学过程，它们之间存在着复杂的关系，并通过多种信号通路介导。EMT过程通过不同的机制在多种癌细胞中产生对细胞死亡的抗性。对治疗有抵抗力或接受EMT的癌细胞可能更容易诱导铁下垂，具有间充质性质的癌细胞通常比具有上皮性质的癌细胞对铁下垂更敏感，这意味着EMT在一定程度上与铁下垂有关^[18]^。

miR-186-5p已被证实与癌症密切相关，我们课题组前期实验研究^[19]^表明，miR-186-5p通过靶向调控PTTG1抑制LUAD细胞的EMT，然而有关miR-186-5p如何通过铁死亡机制影响其在LUAD增殖迁移和侵袭中发挥作用尚未见研究报道，因此，本文通过实验验证敲低PRKAA2能协同miR-186-5p增强LUAD细胞对Fer-1诱导的铁死亡敏感性，逆转Fer-1抑制LUAD细胞铁死亡的趋势，从而抑制LUAD细胞增殖、侵袭、迁移能力，进一步加强证实miR-186-5p在LUAD细胞中所发挥的抑癌作用。

AMPK（5′AMP活化蛋白激酶）作为细胞能量传感器，是多种癌症的关键调节因子^[20]^。PRKAA2是AMPK的α2亚基，在葡萄糖代谢中起重要作用^[21]^。我们的研究表明，PRKAA2是miR-186-5p的直接靶点，敲除PRKAA2促进A549细胞铁死亡，miR-186-5p通过负向调控PRKAA2促进LUAD细胞铁死亡，从而抑制LUAD细胞增殖、侵袭和迁移能力。通过本研究，我们探讨了miR-186-5p促进LUAD细胞铁死亡的分子机制，但关于miR-186-5p通过何种信号转导通路靶向PRKAA2介导LUAD进程的机制尚不清楚，这也将成为我们接下来探索与研究的重点。

**Competing interests**

The authors declare that they have no competing interests.
